# Supplementary material for: Unpredictable disturbance and its effects on activity behavior and lifespan in Drosophila melanogaster
Source: Biol Open. 2025 Jul 22;14(7):bio062071. doi: 10.1242/bio.062071 (PMC12320972; doi:10.1242/bio.062071)
Supplement: Supplementary information [file biolopen-14-062071-s1.pdf]

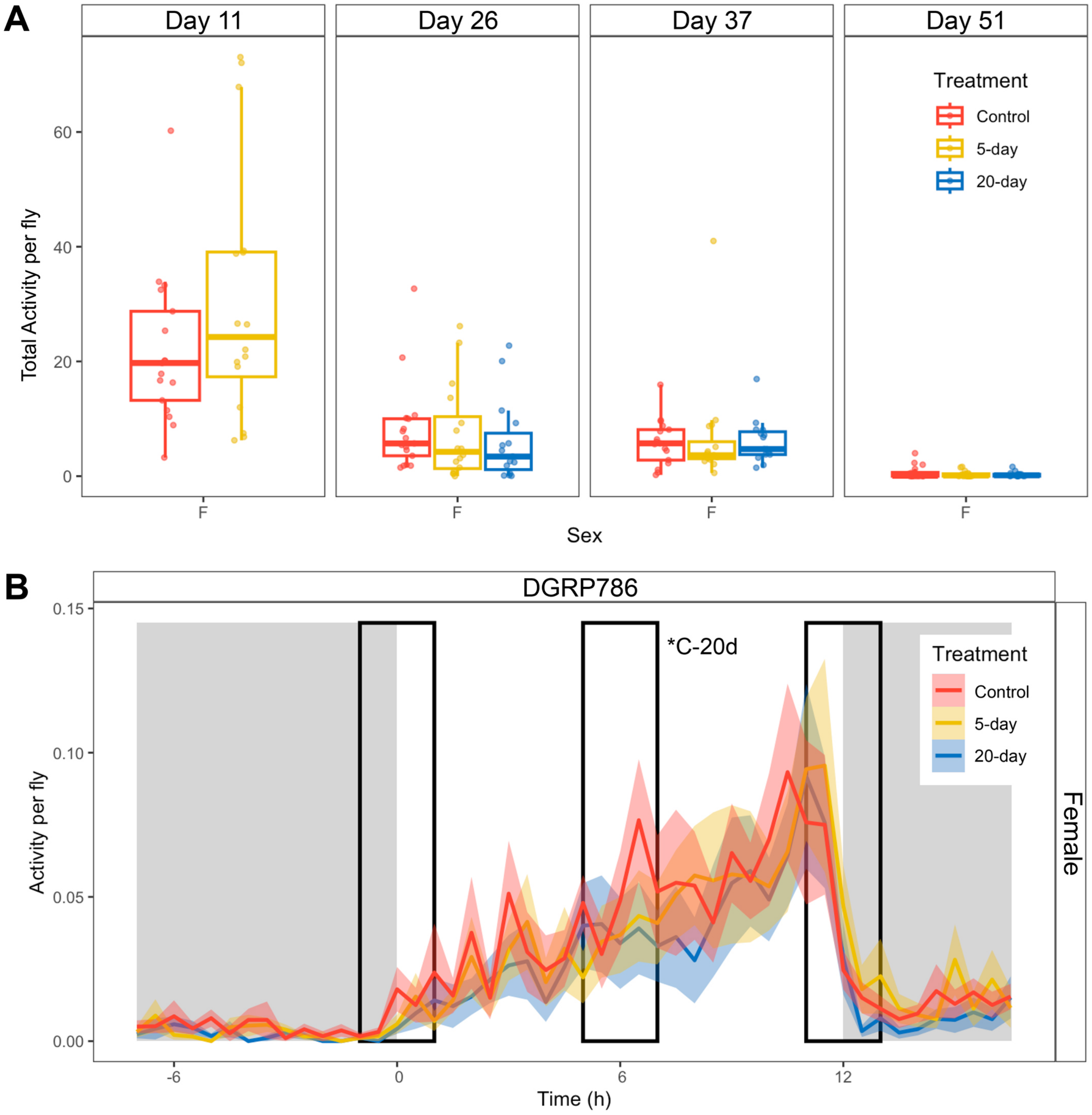

Biology Open • Supplementary information

Fig. S1. Activity data of DGRP786 females.

A. Total activity per fly (y-axis) is plotted against sex (x-axis) for DGRP786 female flies. Activity is measured using the DAMs, which record the number of times flies cross the beam at the midsection of the vial every 5 minutes over 24 hours. Data from the control (red), 5-day disturbance (yellow), and 20-day disturbance (blue) groups show no treatment dependent differences in total activity. The box plots show the 25th and 75th percentile range with whiskers extending to include all data points up to 1.5x the standard deviation. The center line represents median activity. Four ages are plotted: day 11 (n= 16-17 vials/treatment), day 26 (n=16-17 vials/treatment), day 37 (n=16-17 vials/treatment), and day 51 (n=16-17 vials/treatment).

B. Activity per fly (y-axis) is plotted against time in hours (x-axis) for DGRP786 females 26 days old (n=16-17 vials/treatment). Data from the control (red), 5-day disturbance (yellow), and 20-day disturbance (blue) groups show the effect of disturbance on activity. The solid-colored lines represent average activity, and the light shading represents standard error. The grey shaded areas indicate lights off, and the white areas indicate lights on, with time 0 marking lights on at 7AM. The black boxes show activity levels from 6-8AM (left), 12-2PM (middle), and 6-8PM (right). * indicates significant differences ($p<0.05$, pairwise t-test).

Biology Open • Supplementary information

Table S1. Type 3 ANOVA. This dataset has all outliers removed, as this was the best model. Data was removed for DGRP852 on day 51 due to a glitch in the DAMs. Df: degrees of freedom. Gray shading indicates the p-value was significant.

**Day 11**	**Type 3 ANOVA, outliers removed**			
	Sum Sq	Df	F-value	P(>F)
(Intercept)	1475633	1	68.5	4.04e-13
sex	4155551	1	193.0	<2.2e-16
genotype	864259	3	13.4	1.80e-07
sex:genotype	2380365	3	36.9	<2.2e-16
Residuals	2282378	106	-	-
**Day 26**	**Type 3 ANOVA, outliers removed**			
	Sum Sq	Df	F-value	P(>F)
(Intercept)	702839	1	28.8	4.74e-07
sex	8043273	1	329.3	<2.2e-16
genotype	404981	3	5.5	0.0014
sex:genotype	4132830	3	56.4	<2.2e-16
Residuals	2613543	107	-	-
**Day 37**	**Type 3 ANOVA, outliers removed**			
	Sum Sq	Df	F-value	P(>F)
(Intercept)	384156	1	3.6	0.06
sex	10662398	1	99.3	<2.2e-16
genotype	513843	3	1.6	0.19
sex:genotype	4954030	3	15.4	2.05e-08
Residuals	11699600	109	-	-
**Day 51**	**Type 3 ANOVA, outliers removed**			
	Sum Sq	Df	F-value	P(>F)
(Intercept)	145082	1	4.2	0.04
sex	3577733	1	102.8	9.15e-16
genotype	79084	2	1.1	0.33
sex:genotype	1856680	2	26.7	1.69e-09
Residuals	2646135	76	-	-

Biology Open • Supplementary information

Table S2. Type 3 ANOVA summary for all activity data in a mixed-effects model. This dataset has all outliers removed, as this was the best model. Df: degrees of freedom. Gray shading indicates the p-value was significant.

	**Type 3 ANOVA, mixed-effects**		
	Chisq	Df	Pr(>Chisq)
(Intercept)	48.1	1	4.7e-12
sex	136.9	1	<2.2e-16
genotype	40.4	3	7.6e-09
age	23.9	3	2.7e-05
treatment	1.3	2	0.52
sex:genotype	148.3	3	<2.2e-16
sex:age	119.0	3	<2.2e-16
sex:treatment	16.8	2	0.00024
sex:genotype:age	277.8	16	<2.2e-16
